# Electrospun Nanofibre Filtration Media to Protect against Biological or Nonbiological Airborne Particles

**DOI:** 10.3390/polym13193257

**Published:** 2021-09-24

**Authors:** Fabrice N. H. Karabulut, Günther Höfler, Naveen Ashok Chand, Gareth W. Beckermann

**Affiliations:** NanoLayr Ltd., 59 Mahunga Drive, Mangere Bridge, Auckland 2022, New Zealand; naveen.chand@nanolayr.com (N.A.C.); gareth.beckermann@nanolayr.com (G.W.B.)

**Keywords:** nanofibre, electrospinning, face mask, filter media

## Abstract

Electrospun nanofibres can outperform their melt-blown counterparts in many applications, especially air filtration. The different filtration mechanisms of nanofibres are particularly important when it comes to the air filtration of viruses (such as COVID-19) and bacteria. In this work, we present an electrospun nanofibre filter media, FilterLayr^TM^ by NanoLayr Ltd., containing poly(methyl methacrylate)/ethylene vinyl alcohol nanofibres. The outstanding uniformity of the nanofibres was indicated by the good correlation between pressure drop (ΔP) and areal weight with R2 values in the range of 0.82 to 0.98 across various test air velocities. By adjusting the nanofibre areal weight (basis weight), the nanofibre filter media was shown to meet the particle filtration efficiency and breathability requirements of the following internationally accepted facemask and respirator standards: N95 respirator facemask performance in accordance with NIOSH 42CFR84 (filtration efficiency of up to 98.10% at a pressure drop of 226 Pa and 290 Pa at 85 L·min^−1^ and 120 L·min^−1^, respectively), Level 2 surgical facemask performance in accordance with ASTM F2299 (filtration efficiency of up to 99.97% at 100 nm particle size and a pressure drop of 44 Pa at 8 L·min^−1^), and Level 2 filtration efficiency and Level 1 breathability for barrier face coverings in accordance with ASTM F3502 (filtration efficiency of up to 99.68% and a pressure drop of 133 Pa at 60 L·min^−1^), with Level 2 breathability being achievable at lower nanofibre areal weights.

## 1. Introduction

Filtration plays an important role in purifying and decontaminating two of life’s necessities: water and air. Increasing awareness of the links between air quality and human health has led to a rise in the demand for improved personal protection from airborne pollutants and disease-causing microbes. In this respect, electrospun nanofibres (NFs) provide unique capabilities when used as an active protective layer in facemasks. When compared with commonly used filters containing melt-blown (MB) fibres, electrospun NFs provide enhanced protection against airborne particles, bacteria, and viruses such as COVID-19 [1,2,3,4,5,6]. The increased performance is attributed to the different filtration mechanisms, fibre dimensions, and smaller pore size [2,7,8,9].

Electrospinning (ES) is considered the most suitable method for producing polymeric NFs. This is due to the versatility of ES and its ability to use a wide variety of polymers at both lab and production scale [10]. Historically, NFs were not able to be produced in large enough volumes and at a low enough cost to be commercially viable, especially when competing with existing alternatives such as MB fabrics. Worldwide, the NF market is continuing to grow at a steady rate. Recent technological advancements mean that production rates of ES NFs are closing in on those of conventional MB fabrics.

The ES process can be used to generate functional NFs, which can provide enhanced properties as well as higher surface areas when compared with MB fibres. The high surface areas of ES NFs make them particularly suitable for functionalisation for a wide range of applications including filter media, catalysis, super absorbents, scaffolds for tissue engineering and wound dressings, energy storage, and electronics [11]. The ES NFs can be produced at room temperature from a wide range of biopolymers and synthetic polymers in an energy-efficient production process. The ES process requires polymers to be dissolved in a solvent before processing, and the incorporation of antimicrobial, antiviral, biocidal, and virucidal agents into the ES process can be more easily done than for melt-blown fabric production processes [11,12].

ES uses electrostatic forces to draw charged threads from a polymer solution to create NFs. The diameters of these NFs typically range from 10 to 600 nanometres (nm) [11]. The fibre and pore diameters of the electrospun NF filters can be easily controlled and adjusted. Recently, there has been interest in green ES, which involves water-based solutions [13]. Only a few academic groups, including NanoLayr Ltd., have investigated aqueous solution ES and other methods of fabricating green electrospun NFs.

**Figure 1:**
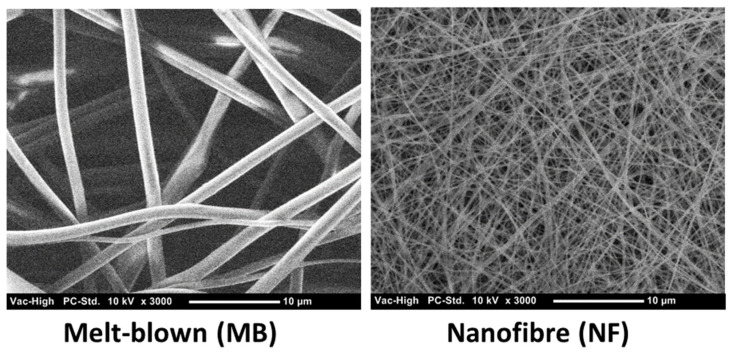
SEM micrographs of melt-blown fibres and ES nanofibres from FilterLayr^TM^.

**Figure 2:**
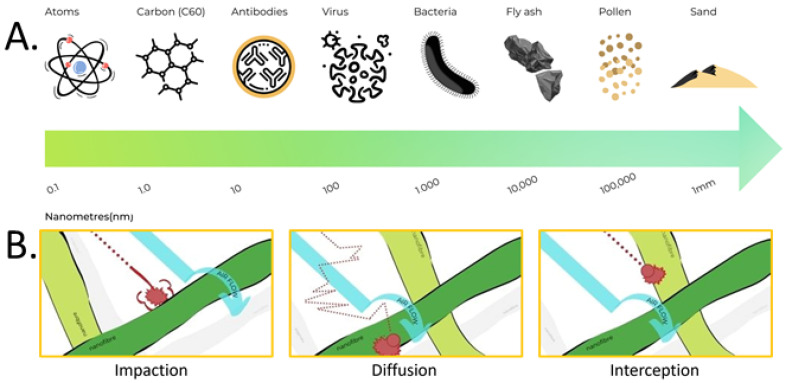
(**A**) Size comparison of particles present in nature; (**B**) the filtration mechanisms associated with electrospun nanofibre filters.

NF filter media produced by ES has attracted much interest in air filtration applications. This is partly due to the fact that the diameters of NFs are 10–100 times smaller than those of conventional MB microfibres (Figure 1). NFs are widely accepted as being particularly effective in stopping submicron and nanometric contaminants with a minimal impact on pressure drop. The higher surface area of NFs induces better filtration efficiency, largely because surface interaction is the dominant driving force in air filtration. ES NFs are dependent on multiple filtration mechanisms, as shown in Figure 2. As such, ES NFs are not affected by loss of electrostatic attraction to the same degree as MB filters. This means that when the electrostatic charge is lost, the filtration efficiency of MB filters drops. Such electrostatic charges can be lost due to moisture in the environment or ageing of the material. In addition, the ES process offers opportunities for the fine tuning of surface functionality through polymer chemistry.

To understand how ES NFs enhance filtration performance, it is important to understand the particle-capturing mechanisms. Particles can be blocked by a filter via five different mechanisms: Sieving, Interception, Inertia Impaction, Diffusion, and Electrostatic Attraction (Figure 2). Gravity can aid the filtration process but is often considered negligible for particles smaller than 600 nm. Particles can be classified in different sizes, as shown in Figure 2. Particles larger than the pore size of the filter are captured by the sieving mechanism. When the filter is charged, oppositely charged particles are attracted and deposited on the filter by electrostatic attraction. Smaller particles not captured by these mechanisms are filtered according to inertial impaction, interception, and diffusion. Inertial impaction works on particles between 300–600 nm [14,15], which follow the airflow. These particles are heavier than the air fluid surrounding them. As the air flow splits in different directions when entering the fibre pore, the particles continue in a straight line, impact, and deposit on the fibre surface. Diffusion is very efficient on the smallest particles (<300 nm) [14,15]. Such particles are not held in place by fluid air and diffuse randomly within the air stream. As the particles traverse the flow stream with random motion, they hit the fibre and are deposited. Direct interception works on particles [14,15] that are not large enough to have inertia and not small enough to diffuse within the airflow stream. These midsized particles follow the air stream as it bends through the fibre spaces. Particles are intercepted when they collide with a fibre.

Due to the various mechanisms by which filtration occurs, the smallest particles are typically not the most difficult to filter out. Most air filters have a region of lower filtration efficiency somewhere between 0.1–0.5 µm [16]. Particles in this range are large enough not to be effectively captured by diffusion, but small enough not to be effectively captured by interception or impaction. The most penetrating particle size (MPPS) will depend on the filter media, air flow rate, and electrostatic charge on the particle. Most toxic particulate compounds are smaller than 1 micrometre in diameter. Conventional mechanical fibrous filters (such as MB filters) remove micrometre-sized particles with high efficiency. However, for particles in the submicron range, ES NF are considered better as they offer enhanced filtration performance. This is due to their high surface area and small pore diameter [17]. Electrospun NFs are characterised by a very large surface area, which significantly increases the probability of the particles depositing on the fibre surface, thereby improving the filter efficiency. In addition, ES NF materials have low areal weights, high permeability, and small pore sizes that make them appropriate for a wide range of filtration applications [18]. ES NF filters contain NFs with smaller fibre diameter (10–500 nm), and smaller and more uniform pore sizes than commonly available MB N95 face masks, which are made of PP fibres with diameters in the range of 800~3000 nm. In general, air filtration is primarily based on depth filtration via the combined effects of sieving, inertial impaction, interception, diffusion, and electrostatic interactions. Figure 2 shows the typical capture efficiency curves for particles captured by fibrous filters as a function of particle diameter [7].

As discussed above, some particles in the nano range (100~500 nm) are difficult to filter as they do not conform to one particular capture mechanism. Filtration of MPPS particles require multiple and uniform layers of NF, which defer the particles so that they obey one of the capture mechanisms. Multilayer filters are often hindered by poor breathability and high pressure drop, which is undesirable for air filtration. However, electrospinning enables control of the porosity, packing density, fibre diameter, and surface area of the NFs. Modification of these parameters allows for customisation of NF air filters to allow for the filtration of a wider range of particles/contaminant types, sizes and concentrations can then be achieved with MB filters. As shown in Figure 2, the smaller the fibre diameter, the higher the overall filtration efficiency for NF filters. Electrospun NFs can thus be optimised for filtration performance and, hence, pressure drop/breathability can also be tuned or optimised.

The recent COVID-19 pandemic is caused by the novel coronavirus, SARS-CoV-2, which is transmitted primarily through direct contact with respiratory droplets of an infected person (generated through coughing and sneezing) [19,20,21]. The best nonpharmaceutical interventions against disease spread via respiratory means are broadly termed social or safe social distancing measures, such as reducing close contact between individuals [19,21,22]. Where safe social distancing is not possible, personal protective equipment (PPE) is the most accepted method of self-protection. Masks and respirators are arguably the most important piece of PPE. There are many different types of face masks and respirators available, each offering different levels of protection to users [19,23,24,25,26,27]. The filtering capacity, and hence, the level of protection against pollutants and pathogens, depends primarily on the materials used as well as the engineering design [23,24,25,26,27]. Contaminants in the air differ vastly in size (Figure 2). The SARS-CoV-2 virus typically ranges from 60 to 140 nm in size [28] and is smaller than bacteria, dust particles, and pollen. Therefore, masks and respirators made of materials with larger pore sizes, such as cotton and synthetic woven and nonwoven fabrics, will not be able to filter these viruses or tiny virus-laden droplets as effectively as those made of materials with much smaller pore sizes such as NFs. The aim of this paper is to investigate the filtration efficiency of NF filter media when tested in accordance with three different international standards: ASTM Test Method F2299 for surgical facemasks, ASTM Test Method D3502 for barrier face coverings, and NIOSH 42CFR84 for respirator masks (N95). In 2019, Akduman reported the fabrication of nanofibrous filter media made of cellulose acetate (CA) and polyvinylidene fluoride (PVDF) and tested different nanofibre areal weights to the NIOSH 42CFR84 (N95) test standard [29]. The author also studied the effect of nanofibre diameter on filtration efficiency and met the requirements of the NIOSH 42CFR84 (N95) test standard for three samples made at different polymer concentrations with different collection times (16%CA–60 min, 15%CA–30 min, and 10%PVDF–15 min). However, the applicability and scale-up of these formulations (16%CA–60 min, 15%CA–30 min, and 10%PVDF–15 min) are still unknown. In this investigation, more than 75 electrospun NF/spunbonded polypropylene filter media samples were produced (15 cm × 15 cm sample sizes), and their filtration performances were challenged against different particle sizes and types at different air velocities. This study describes the influence of NF areal weight on the particle filtration efficiency (PFE) and pressure drop (ΔP) of NF air filter materials when tested in accordance with the three international standards cited above. The production of filter materials containing ES nanofibre can easily be scaled-up by means of NanoLayr’s proprietary Sonic Electrospinning™ process to meet the growing international demand of such materials.

## 2. Experimental

### 2.1. Materials

Various ES solutions were prepared using the following materials: formic acid, acetic acid, ethylene vinyl alcohol (EVOH), and poly(methyl methacrylate) (PMMA) supplied by Merck; spunbonded polypropylene nonwoven substrate fabric (SPP).

### 2.2. Electrospinning of Nanofibre and Characterisation

Electrospinning is the most commonly used technique for the mass-production of polymer NFs and has been explained in detail by Rutledge and Fridrikh [30]. A unique, large-scale, needleless ES process, developed by Nanolayr Ltd. (formerly known as Revolution Fibres Ltd., Auckland, New Zealand), was used to manufacture rolls of NF filter media for this investigation. ES solutions were produced by dissolving a specified quantity of polymer into a suitable solvent, as detailed in Section 2.5. During the ES process, droplets of a polymer solution were applied to the positively charged electrodes of the ES machine. The polymer solution was then drawn and spun through the electrostatic field before being deposited as randomly oriented continuous NFs onto a spunbonded polypropylene substrate (SPP) resting on a negatively charged collector plate. The distance between the electrodes and the collector was set to 110 mm and a voltage differential of 70 kV was applied to draw fibres out of the polymeric solution. The environmental conditions were set to 23 °C and an average relative humidity of 50%. Nanofibre areal weights were determined by weighing 100 cm^2^ samples on a Precisa XB220A analytical balance and then dividing the sample mass by the sample area. The unit of the areal weight is gram per square metre (GSM).

NF samples were analysed using a JEOL JCM-5000 scanning electron microscope (Tokyo, Japan) and fibre diameters were measured using an evaluation software called Fibraquant, whereby the average NF diameters were determined with from 50 to 100 measurement readings. 

### 2.3. Pressure Drop (ΔP) and Breathing Resistance

Pressure drop is the measure of the difference in total pressure between two different points of a fluid (air) as it flows through the filter media. This pressure drop is due to frictional forces caused by the resistance to flow. The pressure drop of different filter media, including samples containing NF at different areal weights, were measured using a TexTest FX 3300 LabAir IV (Schwerzenbach, Switzerland) in accordance with ASTM Test Method F2299.

Breathing resistance or breathability is a measure of the difficulty to breathe in or out through a mask or filter media and is commonly expressed as a pressure drop across the fabric layers. Different filter media including NFs at different areal weights were measured using a PALAS PMFT 1000 (Karlshrue, Germany) in accordance with ASTM Test Method D3502.

### 2.4. Filtration Performance Testing

Particle filtration efficiency (PFE) is a measure of the proportion of particles that are intercepted by the mask or filter material. The general approach to determine the PFE is to challenge the test sample with small particles that are carried in the air and move through the test specimen at a specific airflow velocity (also termed face velocity), and to measure the particle concentration upstream (before) and downstream (after) of the test sample. The ratio between the particle concentration downstream and the particle concentration upstream is the filter penetration (P_filter_ = C_down_/C_up_ × 100%). PFE is the complement of filter penetration (PFE (%) = 100% − P_filter_). A filter media with a PFE of 98% will block 98% of particles (of all particles or at a specific particle size) so that only 2% of particles will pass through the filter media when air is inhaled or exhaled. It should be noted that the values for PFE presented in this paper are measured only on the filter media and not on fabricated masks. The PFE of facemasks may vary during filter testing due to air leakage around the edge of the mask, and it is typically the responsibility of the mask manufacturer to guarantee compliance by further testing of their final product. Different filter media including those containing NFs at various areal weights were measured using a PALAS PMFT 1000 according to NIOSH 42CFR84 (N95), ASTM Test Method D3502, and ASTM Test Method F2299. A comparative summary of the filtration test methods of all three of these international test standards can be seen in Table 1.

### 2.5. Electrospinning Solutions

The PMMA used in this research has an average molecular weight of 120,000, a glass transition temperature of 105 °C, and density of 1.188 g/mL at 25 °C. The EVOH used contains 38 mol% ethylene, has a glass transition temperature of 58 °C, an MFR of 8.0 g/10 min, and a density of 1.17 g/cm^3^ at 23 °C. Electrospinning solutions were prepared by adding PMMA and EVOH granules (15 wt%) slowly into a blend of formic acid (99% purity) and glacial acetic acid and stirred at room temperature until all the particles had fully dissolved. 

## 3. Results and Discussion

**Figure 3:**
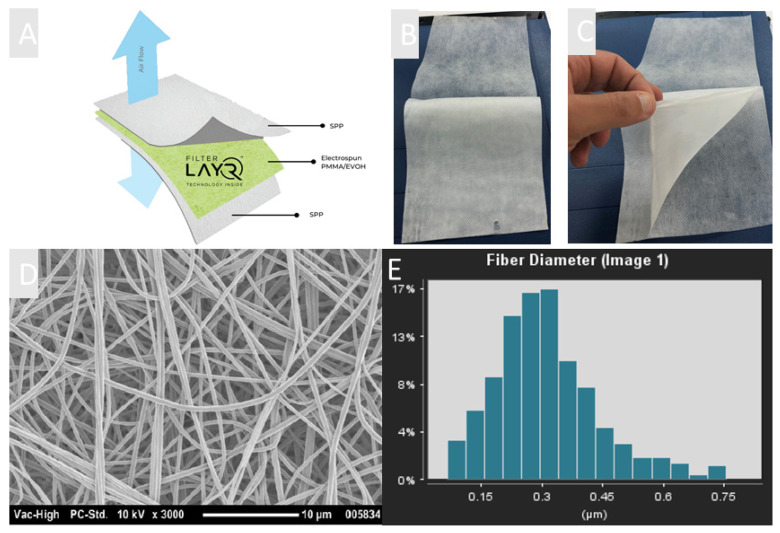
(**A**) Diagram of FilterLayr^TM^ structure: spunbonded polypropylene/PMMA–EVOH electrospun nanofibre/spunbonded polypropylene (SPP/PMMA–EVOH/SPP). (**B**) Macroscopic image of SPP/PMMA–EVOH/SPP structure. (**C**) Nanofibre layer in FilterLayr^TM^. (**D**) SEM micrograph of nanofibre layer made from a blend of PMMA and EVOH. (**E**) Average fibre distribution of PMMA–EVOH electrospun fibres using Fibraquant image analysis software.

NanoLayr Ltd., an AS9100d certified advanced materials manufacturing company based in New Zealand, has developed a unique filter media containing electrospun NF made from blends of PMMA and EVOH. This product, marketed as FilterLayr^TM^, is a 3-layer structure consisting of two outer layers of nonwoven fabric with a middle layer consisting of kilometre-long NFs (Figure 3a–c). Figure 3d,e show SEM images of randomly oriented NFs fabricated from PMMA/EVOH solutions with an average fibre diameter of 318.9 ± 29.2 nm. The morphology of the NF layers shows a typical nonwoven stacking structure. The use of nanoscale fibres has proven to be advantageous in air filtration, since their small diameters and high surface-to-volume ratios can enhance the capture of particles through interception and other mechanisms [31,32,33]. It has also been shown that homogeneous porosity can result in lower pressure drop due to the slip flow effects of the NFs when compared with microfibre’s counterparts [34]. These excellent characteristics make electrospun NFs very attractive. It is known for traditional air filter media that high filtration efficiency is typically only achieved for thicker materials or at higher areal weights. This is not necessarily the case for NF filter media, where a comparatively low areal weight is required for high filtration performance whilst still maintaining a low pressure drop [33,35,36]. In this study, a series of samples was prepared in order to investigate the optimum NF areal weight to achieve the best filtration efficiency whilst still maintaining a low pressure drop (breathing resistance). NFs of different areal weights ranging from 0.65 gsm to 2.1 gsm were electrospun onto a SPP substrate. Thereafter, another top layer of SPP was used as a cover layer to protect the NF and to provide better structural rigidity to the filter (Figure 3). In this investigation, filtration efficiency and pressure drop (breathing resistance) of filter media were measured in accordance with three different international standards.

### 3.1. Pressure drop (ΔP) vs. Areal Weight in GSM

**Figure 4:**
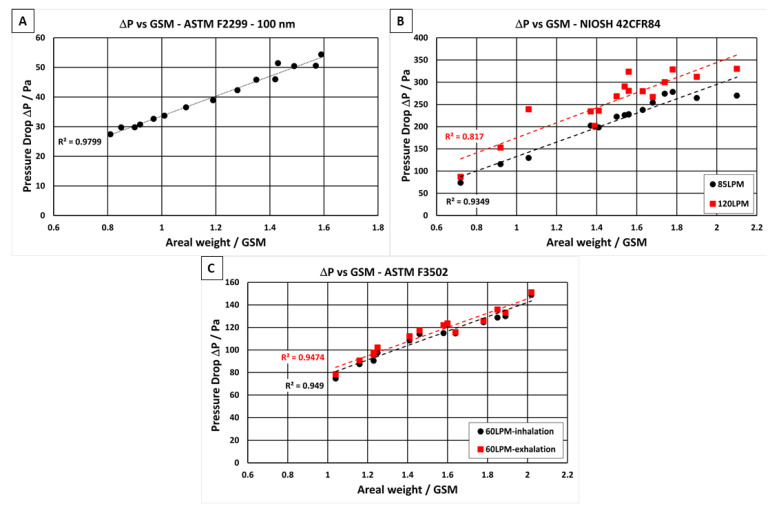
Relationships between pressure drop (ΔP) and nanofibre areal weight (in gsm) when tested in accordance with different international standards: (**A**) ASTM F2299, (**B**) NIOSH 42CFR84, and (**C**) ASTM F3502.

By combining the NF layer with a stronger polypropylene substrate, the presented material has sufficient mechanical strength to be used in filter applications. It has been shown that many commercially available NF filters often have lower filtration efficiencies than what is reported in their design specifications due to the nonuniformity of the NF, especially when the NFs are produced using conventional needle-based electrospinning techniques. This is mostly because the electric field between the conductive needle tip and the grounded substrate surface is nonuniform due to the uneven nature of the substrate fabric [37,38,39,40]; however, there are many other electrospinning variables that can also influence the uniformity of the NF produced, such as voltage differential, humidity, temperature, solution viscosity, and conductivity [41,42]. Nonuniformity of the NF layer is undesirable as it can lead to a high degree of variability in both the filtration efficiency and the pressure drop of the filter media. Kim et al. [43], reported the importance of uniformity of NF filter media to achieve great filtration performance. The authors showed that when the morphology of the NF is not uniform, a dramatic decrease in the overall filtration efficiency of the material is observed. However, advancements in needleless electrospinning techniques at NanoLayr Ltd. have overcome the issues of NF nonuniformity, allowing for the production of hundreds of square meters of consistent NF in a day. In order to show the degree of NF uniformity, the pressure drop of the NF filter media was plotted against the areal weight of NF, as seen in Figure 4. In an ideal situation, the pressure drop and NF areal weight should follow a linear trend, where an increase in NF areal weight results in a predictable increase in pressure drop of the material. The plots in Figure 4 show pressure drop vs. areal weight of the different filter media when tested at three different air velocities, as described in international test standards. NF areal weight, when considered alongside pressure drop, can be used as a measure to understand the packing density of the NF layer in the filter media. For the three standards considered, the pressure drops measured at the four different air velocities (8 L·min^−1^, 60 L·min^−1^, 85 L·min^−1^, and 120 L·min^−1^) increased when the NF areal weight increased from 0.65 gsm to 2.1 gsm. All the samples tested showed a linear relationship between pressure drop and areal weight, with R^2^ values ranging from 0.82 to 0.98 to verify the uniformity of the NF layer in the filter media.

### 3.2. Filtration Performance when Tested to Different International Standards

**Figure 5:**
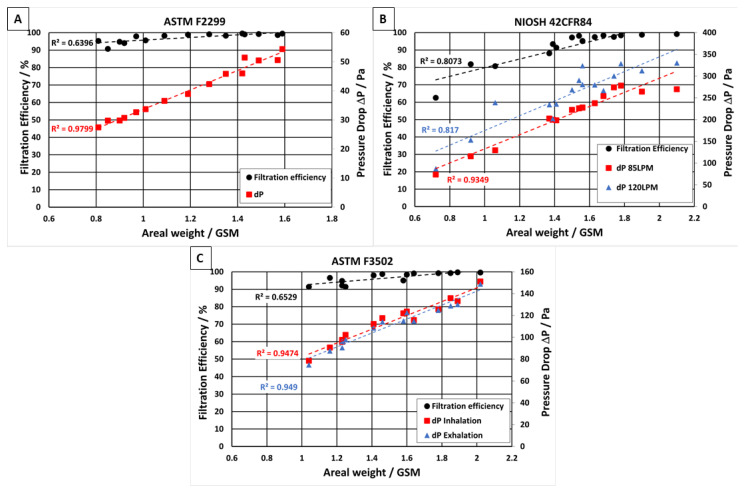
Filtration efficiency and pressure drop vs. NF areal weight (in gsm) when tested in accordance with different international standards: (**A**) ASTM F2299, (**B**) NIOSH 42CFR84, and (**C**) ASTM F3502.

A series of different filter media containing different NF polymer blends were tested in accordance with different international standards; the filtration efficiency and pressure drop results are shown in the supporting information Tables S2 and S3. The filtration efficiency and pressure drop (or breathing resistance) at various NF areal weights can be seen in Figure 5. For these filtration efficiency tests, the samples of filter media were challenged with monodispersed polystyrene latex sphere (PSL) aerosols with an average particle diameter of 0.1 µm. Other samples were challenged with 0.3 µm and 0.5 µm-particle-sized PSL aerosols, see Figure S1 for supporting information. The PLS’ were nebulized, dried, and passed through the filter media at an airflow velocity of 8 L·min^−1^, as required for the PFE test method in ASTM F2299. All filter media samples containing NF layers of more than 1.1 gsm met the particulate filtration requirement for Level 2 ASTM F2299 facemasks and showed filtration efficiencies of ≥98% (Figure 5a) and relatively low pressure drops ranging from 27 to 54 Pa (at air velocities of 8 L·min^−1^). The detailed ASTM F2299 pressure drop and filtration efficiency results at particle sizes of 100 nm, 300 nm, and 500 nm are displayed in Table 2. Table 3 shows ASTM F3502 and NIOSH 42CFR84 filtration efficiency and pressure drop results. In Figure 5b, samples of filter media were challenged with 0.3 µm-sized NaCl particles at air velocities of 85 L·min^−1^ in accordance with the NIOSH 42CFR84 test method. In this case, all filter media samples containing NF layers of more than 1.5 gsm met the N95 facemask filtration efficiency requirements of NIOSH 42CFR84, and all samples showed pressure drops below 330 Pa at 120 L·min^−1^. To pass the standard requirements of both filtration efficiency and breathability, the desired areal weight ranges from 1.50 to 1.63 gsm. For the standard specification for barrier face coverings, ASTM F3502, a minimum filtration efficiency of 20% is necessary to meet the requirements of level 1, and ≥50% filtration efficiency is required to meet the requirements of level 2 (Table 1). Figure 5c illustrates that each of the filter media samples tested exceeded the filtration performance requirements of the ASTM F3502 test method. These results show that filtration efficiency values of up to 90% can be achieved for filter media containing a NF layer greater than 1 gsm. Each of the filter media samples tested in accordance with ASTM F3502 passed the level 1 breathability requirements. However, if a lower NF areal weight were used, it is possible to pass level 2 breathability while keeping the filtration efficiency above 50%.

### 3.3. Filtration Efficiency vs. Particle Size

**Figure 6:**
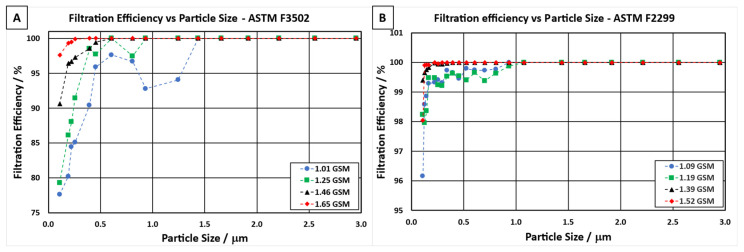
Filtration efficiency of filter media containing different NF areal weights vs. particle size when tested in accordance with different international standards: (**A**) ASTM F3502 and (**B**) ASTM F2299.

The different electrospun NF filter media were further analysed to evaluate their filtration efficiency at various particle sizes, as shown in Figure 6. The graphs indicate a good correlation between an increase in NF areal weight and improved filtration efficiency over the range of particle sizes. For NF filter media tested in accordance with ASTM F3502 (Figure 6a), 100% filtration efficiency of particle sizes 300 nm, 600 nm, 900 nm, and 1400 nm could be attained for NF areal weights of 1.65 gsm, 1.46 gsm, 1.25 gsm, and 1.01 gsm, respectively. The sample with a NF areal weight of 1.01 gsm showed a lower filtration efficiency at larger particle sizes (900 nm and 1400 nm), which can be explained by the different filtration mechanisms discussed earlier in this work. Figure 6b shows that in order for a NF filter media to pass the requirements for ASTM F2299 Level 2, a NF areal weight of over 1.1 gsm is required. Similarly, the higher the NF areal weight in the filter media, the higher the filtration efficiency of smaller particles.

## 4. Conclusions

A novel air filtration media (marketed as FilterLayr^TM^) containing electrospun PMMA/EVOH NFs has been developed by NanoLayr Ltd. NF filters are excellent for use in air filtration applications, since they can achieve enhanced particle capture while offering low pressure drop and homogeneous porosity. In this study, filter media with NF areal weights ranging from 0.65 gsm to 2.1 gsm were analysed using a PALAS PMFT 1000 Filter Test System and a TexTest FX 3300 LabAir IV Air Permeability Tester.

After testing, the pressure drop of sample materials with differing NF area weights were evaluated at various test air velocities, in accordance with each test standard. The R2 values between the pressure drop and areal weight at the different air velocities ranged from 0.82 to 0.98 gsm, indicating excellent uniformity of the NF filter media produced.

With regards to filtration efficiency, NIOSH 42CFR84 N95 was achieved for sample material with NF areal weights of over 1.5 gsm (with a filtration efficiency reaching 98.10%), and ASTM F2299 Level 2 was achieved for samples with NF areal weights exceeding 1 gsm (with a filtration efficiency reaching 99.97% for 300 nm-sized particles and a pressure drop of 44 Pa). All tested samples outperformed the minimum requirements of ASTM F3502 for Level 2 filtration efficiency and Level 1 breathability (with a filtration efficiency of up to 99.68% and a pressure drop below 133 Pa), with Level 2 breathability being achievable only at lower NF areal weights. It was also clear from the results that better filtration efficiencies could be achieved with heavier NF layers, and this was more prevalent with the filtration of smaller aerosol particle sizes. It can be concluded that NanoLayr Ltd. achieved the industrial-scale production of a novel nanofibre filter media, which, unlike other nanofibre media, can meet the filtration efficiency and pressure drop requirements of three major and significantly different international test standards for respirator facemasks, surgical facemasks, and barrier face coverings. There is, however, an increasing need for the development of self-decontaminating facemask and filter media that have the added benefits of being able to inactivate the pathogens, viruses, and bacteria that they capture [44]. Future work will involve the development of functional antimicrobial nanofibre filter media, which can still meet the same international test standards presented in this paper.

## Figures and Tables

**Table 1:** Comparison of filtration test method requirements.

Test		Level 1	Level 2	Level 3
**ASTM F2299 (PFE)-1** *Filtration at 0.1 µm—28.3 L·min^−1^*		95%≤	98%≤	
**EN14683** *(Breathing resistance—Breathability)*		≤49 Pa	≤58.8 Pa	
**ASTM F3502** *Filtration at 0.3 µm—60 L·min^−1^*		20%≤	50%≤	
**ASTM F3502** *(Breathing resistance—Breathability)*		15 mmH_2_O (147.5 Pa)	5 mm H_2_O (49 Pa)	
**NIOSH 42 CFR 84** *Filtration at 0.3 µm—85 L·min^−1^*		**N95** 95%≤		
**Breathing resistance**	Inhalation—120 L·min^−1^	<314 Pa		ΔP < 98 Pa
	Exhalation—85 L·min^−1^	<245 Pa		

**Table 2:** Filtration efficiency and pressure drop results across varying aerial weight tested in accordance with ASTM F2299.

ASTM F2299—100 nm				ASTM F2299—300 nm				ASTM F2299—500 nm			
#	GSM	Filtration Efficiency @100 nm	ΔP	#	GSM	Filtration Efficiency @300 nm	ΔP	#	GSM	Filtration Efficiency @500 nm	ΔP
1	0.81	95.25	27.43	1	0.45	96.11	16.08	1	0.65	98.86	17.11
2	0.85	90.69	29.74	2	0.75	97.11	24.93	2	0.68	99.15	18.47
3	0.9	94.73	29.76	3	0.83	99.35	29.82	3	0.89	99.11	27.11
4	0.92	93.97	30.67	4	1.05	99.2	33.43	4	0.9	99.68	27.58
5	0.97	97.83	32.61	5	1.09	99.87	38.09	5	1.07	99.92	28.36
6	1.01	95.53	33.68	6	1.13	99.9	39.63	6	1.21	99.57	35.12
7	1.09	98.16	36.51	7	1.19	99.48	41.71	7	1.23	99.90	39.05
8	1.19	98.83	38.88	8	1.2	99.85	40.11	8	1.26	99.99	41.33
9	1.28	99.01	42.29	9	1.23	99.97	44.45	9	1.28	99.98	40.68
10	1.35	98.21	45.83	10	1.32	99.94	47.42	10	1.31	99.90	41.41
11	1.42	99.5	45.96	11	1.43	99.7	49.92	11	1.33	99.90	42.81
12	1.43	98.97	51.42	12	1.5	99.96	48.36	12	1.35	100.00	43.32
13	1.49	99.16	50.42	13	1.5	99.99	55.87	13	1.37	99.93	45.86
14	1.57	98.59	50.55	14	1.52	99.83	53.17	14	1.42	100.00	47.12
15	1.59	99.39	54.34	15	1.56	99.98	52.09	15	1.67	100.00	51.84

**Table 3:** Filtration efficiency and pressure drop results across varying aerial weights tested in accordance with ASTM F3502 and NIOSH 42CFR84.

ASTM F3502—300 nm					NIOSH 42CFR84—300 nm				
#	GSM	Filtration Efficiency @300 nm	ΔP Inhalation	ΔP Exhalation	#	GSM	Filtration Efficiency @300 nm	ΔP Inhalation	ΔP Exhalation
1	1.04	91.38	78.40	74.58	1	0.72	62.47	73.65	86.98
2	1.16	96.49	90.55	87.40	2	0.92	81.82	115.63	152.83
3	1.23	94.76	97.52	96.27	3	1.06	80.68	129.40	238.99
4	1.23	92.12	95.71	90.37	4	1.37	88.02	202.35	234.12
5	1.25	91.38	102.07	97.63	5	1.39	93.35	199.60	201.60
6	1.41	97.94	112.08	108.40	6	1.41	91.27	198.22	235.74
7	1.46	98.64	117.46	114.08	7	1.5	97.17	222.31	268.26
8	1.58	94.98	121.90	114.86	8	1.54	98.10	225.76	290.21
9	1.6	98.37	123.50	122.45	9	1.56	95.18	227.13	280.45
10	1.64	99.03	115.66	114.67	10	1.56	95.06	227.82	323.54
11	1.78	99.16	125.65	124.62	11	1.63	97.40	237.46	279.64
12	1.85	99.20	135.81	128.70	12	1.68	98.33	253.98	266.63
13	1.89	99.68	133.05	129.91	13	1.74	97.46	273.94	299.96
14	2.02	99.54	151.02	148.74	14	1.78	98.37	278.07	328.41
					15	1.9	98.72	264.30	312.15
					16	2.1	99.11	269.81	330.04

## Data Availability

Not applicable.

## Supplementary Materials

The following are available online at https://www.mdpi.com/article/10.3390/polym13193257/s1, Figure S1: Relationships between pressure drop (DP) and nanofibre areal weight (gsm) when tested in accordance with ASTM F2299 at a particle size of (**A**) 300 nm and (**B**) 500 nm. Table S1: Experimental data measuring nanofibre filter media samples in accordance with ASTM F2299. Table S2: Experimental data measuring nanofibre filter media samples in accordance with ASTM F3502. Table S3: Experimental data measuring nanofibre filter media samples in accordance with NIOSH 42CR84.
