# Supplementary material for: Electrospun Nanofibre Filtration Media to Protect against Biological or Nonbiological Airborne Particles
Source: Polymers (Basel). 2021 Sep 24;13(19):3257. doi: 10.3390/polym13193257 (PMC8511993; doi:10.3390/polym13193257)
Supplement: Supplementary file 1 [file polymers-13-03257-s001.zip › polymers-1375369-supplementary.pdf]

Supporting information: Electrospun Nanofibre Filtration Media to Protect Against Biological or Nonbiological Airborne Particles

Fabrice N. H. Karabulut [a], Günther Höfler [a], Naveen Ashok Chand [a] and Gareth W. Beckermann [a]

[a] NanoLayr Ltd. 59 Mahunga Drive, Mangere Bridge, Auckland 2022, New Zealand

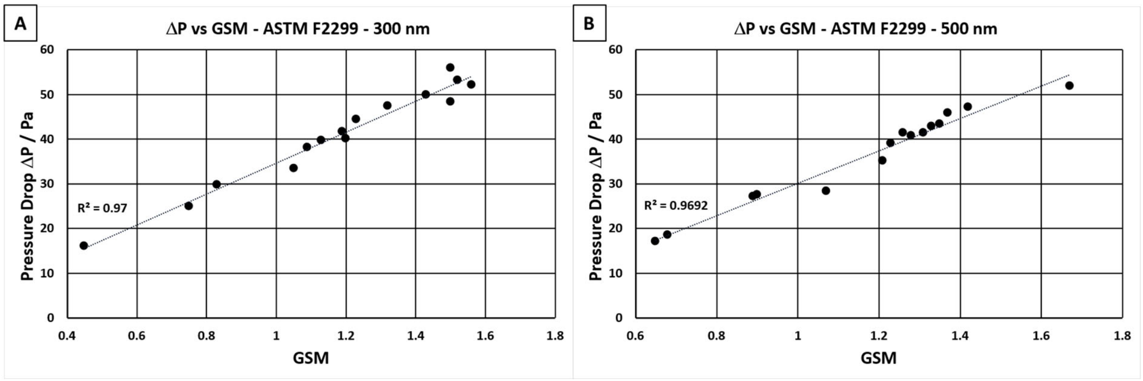

Figure S1. Relationships between pressure drop (ΔP) and nanofibre areal weight (gsm) when tested in accordance with ASTM F2299 at a particle size of (A) 300nm and (B) 500nm.
